# Developmental defects of enamel in children born preterm

**DOI:** 10.3389/fped.2022.1019586

**Published:** 2022-10-13

**Authors:** Elinor Halperson, Salome Shafir, Avia Fux-Noy, Diana Ram, Smadar Eventov-Friedman

**Affiliations:** ^1^ Department of Pediatric Dentistry, Hadassah Medical Center and Faculty of Dental Medicine, Hebrew University of Jerusalem, Jerusalem, Israel; ^2^ Department of Neonatology, Hadassah Medical Center and Faculty of Medicine, Hebrew University of Jerusalem, Jerusalem, Israel

**Keywords:** enamel, preterm, teeth, surfactant, ventilation

## Abstract

**Introduction:**

To investigate manifestations of developmental defects of enamel (DDE) in children born preterm (PT), and to explore possible neonatal morbidities related to DDE manifestation and severity.

**Methods:**

A cohort study of 52 children born before gestational week 32 and treated in the neonatal intensive care unit; and 55 children born at full term (FT) as a control group. All the children had a dental examination at age 1–4 years by a professional pediatric dentist. DDE was defined as an alteration in the enamel surface.

**Results:**

DDE were observed in 23 (44%) and 6 (11%) children, in the PT and FT groups, respectively, odds ratio (OR) = 6.47. The OR for damaged anterior teeth was 12.87 times higher in the PT group. DDE of molars was diagnosed in 19% and 11% of the respective groups. In the PT group, the OR of DDE was 4.1 higher among those with than without respiratory distress. The risk for DDE was 5.7 higher in those who received surfactant than in those who did not. Ventilation length, both invasive and non-invasive, was significantly related to DEE.

**Conclusions:**

DDE was higher in children born PT than FT. The DDE rate was lower than expected based on current literature, and considering the overall increase in survival; this suggests improvement in treatments affecting DEE. Respiratory distress syndrome, surfactant administration reflecting the need for intubation, longer ventilation and local oral trauma were risk factors for DDE. We recommend routine dental examinations in follow up of children born PT, particularly those exposed to assisted ventilation.

## Introduction

Continuous improvement in neonatal medicine over the past two decades has resulted in increased survival of newborns, together with attempts to decrease morbidities. Enhanced quality of life, including oral health, has become one of the most important challenges in modern neonatology (1, 2).

The primary teeth start to mineralize between the 13th and 16th week of gestation and continue development and maturation during the first year of life. Developmental defects of enamel (DDE) are defined as aberrations in the quality and quantity of dental enamel, and are caused by disruption or damage to the enamel organ during amelogenesis (3, 4). The prevalence of DDE in the primary dentition has been reported at rates ranging between 13% and 45%, and can reach 46% to 96% in children born preterm (PT) (5–18). The defects are usually located on the primary teeth, which undergo mineralization of the primary incisors, canines, and first molars; although the second primary molars may also be involved (5). Generalized dental defects, which are usually symmetrically distributed, are most likely due to multifactorial causes, including systemic illnesses associated with birth prematurity, such as low birth weight, respiratory distress syndrome (RDS), mineral deficiencies including hypocalcemia, hyperbilirubinemia, infections during fetal life and the neonatal period, and malnutrition (5–7). Localized defects are most likely caused by laryngoscopy and orotracheal intubation, which are often required by PT infants to overcome lung immaturity and respiratory distress (5). DDE may affect aesthetics and the loss of a protective layer, increase dental plaque retention, and predispose teeth to dental decay (10).

Considering the improvement in neonatal medical care during the past decade, and the paucity of data on the prevalence of DDE in the primary dentition of PT infants during this period, we aimed to investigate the prevalence and contributing variables of DEE in children born PT.

## Methods

### Study population

The study population included children born PT, under 32 weeks gestation between the years 2015 and 2018, who were treated after birth in the neonatal intensive care unit at Hadassah Hospital - Hebrew University Medical Center, Jerusalem, Israel. Parents were contacted and the children were invited to a thorough dental examination at the Pediatric Dentistry Department at Hadassah Medical Center, as part of neonatal follow up. The study inclusion criteria were children born PT, at less than 32 weeks of gestation, who survived. The children were examined between age 2 and 4 years. Exclusion criteria included infants with major congenital malformations, syndromes, and genetic diseases or children using routinely medications associated with dental developmental defects. In addition, children with dental trauma or major decay that compromised tooth crown integrity were excluded. The control group included children born at term (37 weeks and above) with birth weight >2500 g, who were patients at the Department of Pediatric Dentistry (age 2 to 4 years) and were arbitrarily selected.

### Data collection

The following data were collected from a questionnaire filled in by the parents: gender, age, general health condition, pregnancy course, multiple pregnancy, week of birth, birth weight, type of birth, intubation at birth, oral hygiene habits, prolonged bottle use or breast feeding (longer than one year), oral habits, and diet.

In addition to the questionnaire, for the PT group, pre- and postnatal data were obtained and confirmed from hospital records. The clinical data included medications given at birth and during hospitalization, RDS, bronchopulmonary dysplasia (BPD) and severity, patent ductus arteriosus (PDA), sepsis, necrotizing enterocolitis (NEC), any grade of intraventricular hemorrhage (IVH), periventricular leukomalacia, seizure during hospitalization, the highest bilirubin level, need for blood exchange, metabolic bone disease of prematurity, and retinopathy of prematurity. The treatment data included the assisted ventilation mode, the need for intubation during hospitalization, intubation duration, days of noninvasive ventilation, surfactant administration, ventriculoperitoneal shunt, hospitalization days, and treatment for metabolic bone disease of prematurity. All intubations were performed orally according to the neonatal intensive unit protocol.

### Dental examination

All the participants were examined by a professional pediatric dentist. The examinations took place in the dental chair at the dental clinic, and were performed by means of a mouth mirror and a dental explorer, without radiograph. Dental examiners were blinded to neonatal course and risk factors. The clinical examination included: soft tissue condition, caries status, and dental enamel defects. Every tooth and surface were examined, and the severity and extent of each defect were recorded in a comprehensive chart. The presence of DDE was categorized according to the extent of defect proposed by Clarkson and Omullane in 1989 (11). Teeth with no defects were scored 0, hypoplastic defects were scored as 1. Enamel hypoplasia was diagnosed when an alteration in the enamel surface was identified. Hypoplasia defects were scored for the incisors and canine by the extent of the defect on the crown - less than 1/3 of the crown = 2, at least 1/3 and less than 2/3 = 3, at least 2/3 = 4. Any hypoplasia defect in the molars was scored as 4. In addition to the clinical record, clinical photographs of patients' front teeth were taken to reexamine.

### Statistical analysis

All data were inserted into Excel software and calculations were performed using SPSS statistical software (SPSS, version 25.0, IBM, NY). Quantitative variables were described as means and standard deviations, and as medians and ranges. Qualitative variables were presented by frequencies and percentages. Differences were examined between study groups in sociodemographic, birth, clinical, and treatment data. To compare quantitative variables, the ANOVA test was used; and for variables not normally distributed, the Kruskal-Wallis or Mann-Whitney test. Categorical data were compared by using the chi test or Fisher's test.

## Results

### Study population

Of 316 children previously born preterm during 2015 and 2018, 56 arrived to dental examinations. The remaining 260 children did not participate due to the following reasons: not-updated phone number, living in far districts, or had a recent dental checkup. Four were excluded according to the exclusion criteria. The control group included 55 children born at term. Therefore, in total, 107 children participated in the study, 52 children who were born PT and 55 who were born at FT. Characteristics of the study population are described in Table 1. The median age at dental examination was younger for the PT than the FT group: 32 vs. 38 months, *p* < 0.03. The PT group comprised 31 boys (60%) and 21 girls (40%); the FT group comprised 21 boys (38%) and 34 girls (62%) (*p*-value 0.02).

**Table 1:** Study population.

	Preterm	Full term	*p* value
Gender			
Males (%)	31 (60)	21 (38)	0.02
Age at dental examination (months)			
Median (range)	32 (19–52)	38 (18–51)	*p* < 0.03
Mean ± S.D	33 ± 9	37 ± 8	* *
Mode of delivery			
Vaginaly (%)	11 (21)	50 (91)	0.00
Gestation age (week)			
Median (range)	29 (24–31)	40 (38–42)	0.00
Mean ± S.D	29 ± 2.0	40 ± 1.1	
Birth weight (grams)			
Median (range)	1198 ± 361	3,275 (2120–4500)	0.00
Mean ± S.D	1,175 (480–1890)	3266 ± 443	
Multiple pregnancy, (Yes,%)	24 (47)	0 (0)	0.00
Intubation at birth (Yes,%)	13 (25)	No intubation	NA
Intubation duration (days)			
Median (range)	1 (0–61)	No intubation	NA
Mean ± S.D	3.94 ± 9.1		
Non-invasive ventilation (days)			
Median (range)	6 (0–91)	No intubation	NA
Mean ± S.D	14.2 ± 20.5		
RDS (Yes,%)	25 (49)	0	NA
Surfactant administration (Yes,%)	27 (53)	0	NA
BDP (Yes,%)	32 (63)	0	NA
PDA (Yes,%)	39 (76)	0	NA
Sepsis (Yes,%)	4 (8)	0	NA
IVH (Yes.%)	36 (71)	0	NA
NEC (Yes,%)	5 (10)	0	NA
Highest bilirubin level			
Median (range)	8.9 (4–16)	NA	NA
Hospitalization days			
Median (range)	65 (30–273)	NA	NA
Treatment for metabolic bone disease of prematurity (Yes, %)			
	7 (14)	0	NA
VP shunt (Yes,%)	3 (6)	0	NA
PVL (Yes,%)	4 (8)	0	NA
Seizure during hospitalization (Yes,%)	3 (6)	0	NA
Retinopathy of prematurity (Yes,%)	5 (10)	0	NA

RDS, respiratory distress syndrome; BDP, bronchopulmonary dysplasia; PDA, patent ductus arteriosus; IVH, intraventricular hemorrhage; NEC, necrotizing enterocolitis; VP, ventriculoperitoneal; PVL, periventricular leukomalacia; NA, non-applicable.

### Developmental defects of tooth enamel

**Figure 1:**
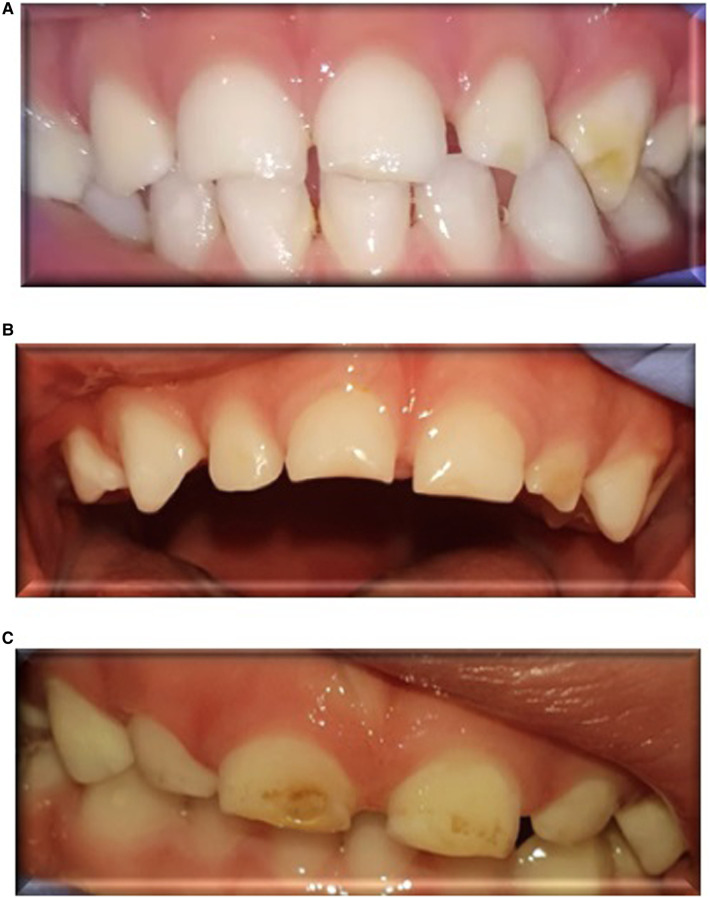
Clinical manifestations of developmental defects of enamel (DDE) in the front teeth of children born preterm. (**A**): DDE in the maxillary left lateral incisor and canine of a 34-month-old boy, born preterm at 25 gestational weeks, who had respiratory distress syndrome at birth, received surfactant, and was intubated for 2 days. (**B**): DDE in the central maxillary incisors and lateral left incisor of a 34-month-old boy, born preterm at 27 gestational weeks, who had respiratory distress syndrome at birth, received surfactant, and was intubated for 9 days. (**C**): DDE in the central maxillary incisors of a 31-month-old girl, born preterm at 30 gestational weeks, who had respiratory distress syndrome at birth, received surfactant, and was intubated for 3 days.

Figure 1 presents clinical DDE manifestations that were observed in the front teeth of children born PT. Table 2 presents data of DDE in children born PT and at FT. Twenty-seven percent of all the children had DDE: 24 (44%) of the PT group and 6 (11%) of the FT group (*p* < 0.00), OR = 6.47.

**Table 2:** Developmental defects of tooth enamel in children born preterm and at full term.

		PT + FT	PT	FT	*P* value	OR
Children with DDE	Number (%)	29 (27)	23 (44)	6 (11)	*p* < 0.00	6.47
Children with DDE in front teeth	Number (%)	19 (17.8)	17 (32.7)	2 (3.6)	*p* < 0.00	12.87
Children with DDE in molars	Number (%)	16 (15)	10 (19)	6 (11)	*p* < 0.40	
Number of teeth with DDE	Mean ± S.D	0.69 ± 1.64	1.13 ± 2.03	0.27 ± 1.00	*p* < 0.00	
Number of front teeth with DDE	Mean ± S.D	0.4 ± 1.10	0.75 ± 1.46	0.07 ± 0.37	*p* < 0.00	
Number of molar teeth with DDE	Mean ± S.D	0.15 ± 0.36	0.2 ± 0.40	0.1 ± 0.31	*p* > 0.80	

PT, preterm; FT, full term; DDE, developmental defects of enamel; OR, odds ratio.

In total, 17.8% of the children had DDE in the front teeth: 32.7% of the PT group and 3.6% of the FT group; OR = 12.87. The proportion of children with DDE in molars did not differ significantly between the groups: 19% vs. 11% (*p* < 0.40). The mean number of damaged teeth, calculated from those that erupted was significantly higher in the PT than the FT group: 1.13 vs. 0.27 (*p* < 0.00). The difference was even more prominent in the front teeth, 0.75 vs. 0.07. A higher mean number of defects was found in molars of the PT than the FT group: 0.2 vs. 0.1, but this difference was not statistically significant.

We analyzed associations of several variables related to neonatal morbidity, with DDE. The analysis included 52 children born PT, of whom 23 (44%) had a tooth defect. Medical information regarding hospitalization was completely available for 51 in the PT group. Only respiratory distress syndrome and surfactant administration were found significant. Twenty-six children had RDS; of them, 16 (61%) had DDE. Accordingly, among children born PT, the OR for DDE was 4.1 (95% CI 1.3–13.3), *p* value < 0.001 among those with RDS compared to those without.Twenty-four children born PT were treated with surfactnat; of them, 16 (67%) had DDE. Accordingly, among children born PT, the OR for DDE was 5.7 (95%CI 1.7–19.1), and the *p*-value was 0.03 for those who received surfactant compared to those who did not.

Table 3 shows ORs for the PT group for the risk of DDE in incisor teeth. Seventeen of 52 (32.6%) children born PT had defects in their incisors. Among children born PT, the OR of DDE in the incisor teeth was 11.2 times higher among those who did than did not receive surfactant. The OR of damaged incisor teeth was 8.55 times higher in those with than without RDS. For the PT group, the mean duration of invasive ventilation was 6.8 days and the mean duration of noninvasive ventilation was 23.3 days for those with DDE; and 2.5 days and 9.8 days, respectively, for those without DDE. These differences between those with and without DDE were statistically significant.

**Table 3:** The risk of developmental defect of enamel in incisor teeth according to various parameters.

Variable		DDE	Healthy teeth	OR	CI 95%	*P* value
Surfactant	Yes (%)/No (%)	14 (58)/3 (11)	10 (42)/24 (89)	11.2	2.6–47.9	0.00
RDS	Yes (%)/No (%)	14 (54)/3 (12)	12 (46)/22 (88)	8.55	2.0–13.3	0.00
Intubation duration (days)	Median (range)	3 (0–61)	0 (0–22)			0.03
	Mean ± S.D	6.82 ± 14.23	2.5 ± 4.66			
Noninvasive ventilation (days)	Median (range)	12 (0–91)	3.5 (0–68)			0.02
	Mean ± S.D	23.29 ± 27.53	9.76 ± 14.22			

DDE, developmental defect of enamel; RDS, respiratory distress syndrome.

At the time of examination, molars had not yet erupted in two children born PT. Ten (20%) of the remaining 50 children born PT had DDE.

## Discussion

Our findings showed a higher prevalence of DDE in the primary dentition of children with PT than children born at FT. This information is worrisome due to the overall increasing survival of extremely PT infants. A meta-analysis published in 2020 showed a three-time increased risk of developing DDE in children born PT (12). Particularly, we found a prevalence of 44% of DDE among children born PT, compared to 11% of children born at FT. However, our rate for DDE in the PT group is on the lower range published in the past two decades (between 46% and 96%) (5–15). Nevertheless, in light of improvements in neonatal care and higher survival of severely PT newborns, an increase in the prevalence of DDE should have been expected. This is mostly important as teeth are true mineral deposition tissue, and alterations caused by neonatal diseases persist in the primary dentition (14). It should be emphasized that drinking water in our population is not fluoridated, as water fluoridation was discontinued in Israel since 2014. Currently the recommended optimal water fluoride concentration ranges between 0.7 and 1.2 parts per million (ppm) depending on the local temperature and water intake amount. No fluoride interventions were performed in the study population or in the control group**.** Thus, we can assume that dental fluorosis is not the cause for the DDE appearance.

The mean number of damaged teeth was also significantly higher in the PT than the FT group: 1.13 vs. 0.27. This concurs with a report published in 2020 that found similar prevalence of teeth damage (1.93 and 0.38, respectively) (18). Those results are significantly lower than prevalences (7.6 and 1, respectively) published two decades ago by Lai PY, et al. (13). The decrease in the prevalence of damaged teeth in children born PT may be contributed to overall improvement in neonatal care.

Differences in DDE between children born PT and at FT varied according to tooth type. DDE in molars were similar between children born PT and at FT. On the other hand, the mean number of incisors with DDE was significantly higher among children born PT than at FT group: 0.75 vs. 0.07. Moreover, 32% of children born PT had an incisor affected by DDE, compared to only 17% of children born at FT. This indicates 12.87 times greater odds of DDE in an incisor of a child born PT than at FT. These findings corroborate previous reports of incisors as the teeth most affected by DDE in children born PT (6, 18). The differences in DDE prevalence according to tooth location suggest local rather than systemic causes. An example of a local effect of traumatic forces in the anterior teeth of children born PT is the use of a laryngoscope during tracheal intubation. In contrast, systemic factors would affect incisors and molars. There are no gender differences reported in our study or in the literature regarding DDE.

Our results suggest strong associations between DDE in children born PT and four of the perinatal variables examined. First, RDS was statistically associated with DDE. Among children born PT, RDS conferred an overall increased risk of DDE, by 4.1 times, and an increased risk for DDE in an incisor tooth, by 8.6. Similar findings were reported by Franco et al. (6). Moreover, among children born PT, receiving surfactant for RDS conferred an overall increased risk of DDE of 5.7, and an increased risk for DDE in an incisor tooth, by 11.2 times. To our knowledge, this association is firstly described. During the surfactant administration involved endotracheal intubation and at least short-duration mechanical ventilation. Therefore, RDS and surfactant administration in our study probably reflect intubation attempts. Previous studies have also shown a strong association between tracheal intubation and DDE (19).

In children born PT, dental development is compromised by derangements of calcium metabolism and other systemic factors. This is compounded by the forces exerted by the laryngoscope in traumatic swing maneuvers during direct laryngoscopy on the oral mucosa at the critical period of amelogenesis in children born PT (14, 19). These complications can be avoided if less aggressive, more accurate technique is used during laryngoscopy for tracheal intubation.

In addition to the above, the duration of assisted ventilation, both invasive and non-invasive ventilation, were significantly associated with increased risks of DDE in the incisors of children born PT. Among children born PT, durations of ventilation, both invasive and non-invasive, were longer among those with DDE in the incisors (mean 6.82 ± SD and 23.29 ± SD days, respectively) than among those without DDE (mean 2.50 ± SD and 9.76 ± SD days, respectively). These findings are in accordance with other studies (15, 19). In the current study, the correlation between DDE and PT was specifically pronounced in the incisors, and we assume that the cause is related to local trauma from intubation. Children who required a longer period of invasive or noninvasive ventilation were more severely affected by neonatal complications involving lung malfunction, such as RDS. The study included PT infants born during 2015 and 2018. During this time period most infants who had RDS received surfactant *via* endotracheal intubation. Nowadays, optional techniques of less invasive surfactant administration (LISA) or minimal invasive surfactant treatment (MIST) are available in the treatment of preterm infants, which minimize the need for intubation albeit do not abolish the use of laryngoscopy (20, 21).

Interestingly, DDE causes greater retention of biofilm and higher adherence of Streptococcus mutans on the wrinkled enamel surface. *Streptococcus mutans* is the main known bacteria which uses carbohydrate and produces acid that cause tooth remineralization of the enamel which leads to dental caries (22, 23). In addition, DDE may result in increased dental sensitivity and may have aesthetic implications.

## Limitations

Our study has several limitations. First, as a retrospective observational study, it cannot establish a causal link between PT and DDE. Second, only 52 (18%) of the invited children arrived to the dental examinations. These data represent a selection bias, as parents who came to the dental clinic might have noticed a visible problem with their children's teeth. Thus, our data may have overestimated the prevalence of DDE. The mean age at the dental examination was lower in the PT than the FT group. This is probably related to the arbitrary selection process of the latter, by which children visited the clinic according to their parents' initiative, in contrast to the children in the PT group, who were actively invited to the clinic. In some of the children born PT, the second molars were not yet erupted when examined. Evaluation at a more advanced age could reveal a higher injury frequency in molars.

We have not recorded the number of attempts in each PT infant. As mentioned, all intubations were orally performed. We can assume that for each intubation at least one attempt was made. Finally, our study was conducted in a single center, and our findings may cannot be generalized to all children born PT.

## Conclusions

We found lower rates of DDE than expected from the increased rate of survival of PT infants, and from data reported in the literature. Overall, this demonstrates improvement in neonatal treatment. The variables that were highly associated with DDE, including RDS, surfactant administration and ventilation length suggest local oral trauma as the main cause. In light of these findings, we recommend on close dental follow up of children born PT, and call for less traumatic use of the laryngoscope, when possible, to further decrease the risk for DEE in this vulnerable population.

## Data Availability

The original contributions presented in the study are included in the article/Supplementary Material, further inquiries can be directed to the corresponding author/s.
